# Clinical Behavior of Breast Cancer in Young *BRCA* Carriers and Prediagnostic Awareness of Germline *BRCA* Status

**DOI:** 10.1200/JCO-24-01334

**Published:** 2025-02-24

**Authors:** Matteo Lambertini, Eva Blondeaux, Loredana M. Tomasello, Elisa Agostinetto, Anne-Sophie Hamy, Hee Jeong Kim, Maria Alice Franzoi, Rinat Bernstein-Molho, Florentine Hilbers, Katarzyna Pogoda, Hans Wildiers, Jyoti Bajpai, Michail Ignatiadis, Halle C.F. Moore, Ann H. Partridge, Kelly-Anne Phillips, Angela Toss, Christine Rousset-Jablonski, Carmen Criscitiello, Tiphaine Renaud, Alberta Ferrari, Shani Paluch-Shimon, Robert Fruscio, Wanda Cui, Stephanie M. Wong, Claudio Vernieri, Kathryn J. Ruddy, Maria Vittoria Dieci, Alexios Matikas, Mariya Rozenblit, Cynthia Villarreal-Garza, Laura De Marchis, Fabio Puglisi, Kenny A. Rodriguez-Wallberg, Francois P. Duhoux, Luca Livraghi, Marco Bruzzone, Luca Boni, Judith Balmaña

**Affiliations:** ^1^ Department of Internal Medicine and Medical Specialties (DIMI), School of Medicine, University of Genova, Genoa, Italy; ^2^ Medical Oncology Department, U.O.C. Clinica di Oncologia Medica, IRCCS Ospedale Policlinico San Martino, Genoa, Italy; ^3^ U.O. Epidemiologia Clinica, IRCCS Ospedale Policlinico San Martino, Genoa, Italy; ^4^ Section of Medical Oncology, Department of Precision Medicine in Medical, Surgical and Clinical Care (Me.Pre.C.C), University of Palermo, Palermo, Italy; ^5^ Medical Oncology Department, Institut Jules Bordet, Université Libre de Bruxelles (U.L.B.), Hôpital Universitaire de Bruxelles (HUB), Brussels, Belgium; ^6^ Department of Medical Oncology, Universite Paris Cité, Institut Curie, Paris, France; ^7^ Division of Breast Surgery, Department of Surgery, Asan Medical Center, University of Ulsan College of Medicine, Seoul, South Korea; ^8^ Cancer Survivorship Program—Molecular Predicitors and New Targets in Oncology, INSERM Unit 981, Gustave Roussy, Villejuif, France; ^9^ Susanne Levy Gertner Oncogenetics Unit, The Danek Gertner Institute of Human Genetics, Sheba Tel Hashomer Medical Center, Affiliated to Tel Aviv University, Tel Aviv, Israel; ^10^ Department of Molecular Pathology, Netherlands Cancer Institute (NKI), Amsterdam, the Netherlands; ^11^ Department of Breast Cancer and Reconstructive Surgery, Maria Sklodowska-Curie National Research Institute of Oncology, Warsaw, Poland; ^12^ Department of General Medical Oncology and Multidisciplinary Breast Center, Leuven Cancer Institute, University Hospitals Leuven, Leuven, Belgium; ^13^ Tata Memorial Centre, Homi Bhabha National Institute (HBNI), Mumbai, India; ^14^ Department of Hematology and Medical Oncology, Cleveland Clinic Taussig Cancer Institute, Cleveland, OH; ^15^ Department of Medical Oncology, Dana-Farber Cancer Institute, Boston, MA; ^16^ Department of Medical Oncology, Peter MacCallum Cancer Centre, Melbourne, VIC, Australia; ^17^ Sir Peter MacCallum Department of Oncology, The University of Melbourne, Melbourne, VIC, Australia; ^18^ Centre for Epidemiology and Biostatistics, School of Population and Global Health, The University of Melbourne, Melbourne, VIC, Australia; ^19^ Department of Oncology and Haematology, Azienda Ospedaliero-Universitaria Policlinico di Modena, Modena, Italy; ^20^ Department of Medical and Surgical Sciences, University of Modena and Reggio Emilia, Modena, Italy; ^21^ Department of Surgery, Leon Berard Cancer Center, Lyon, France; ^22^ Department of Oncology and Hemato-Oncology, University of Milan, Milano, Italy; ^23^ Division of New Drugs and Early Drug Development for Innovative Therapies, European Institute of Oncology, IRCCS, Milan, Italy; ^24^ Cancer Genetics Unit, Bergonie Institute, Bordeaux, France; ^25^ Hereditary Breast and Ovarian Cancer (HBOC) Unit and General Surgery 3—Senology, Surgical Department, Fondazione IRCCS Policlinico San Matteo, Pavia, Italy; ^26^ University of Pavia, Pavia, Italy; ^27^ Breast Oncology Unit, Sharett Institute of Oncology, Hadassah University Hospital, Jerusalem, Israel; ^28^ Faculty of Medicine, Hebrew University, Jerusalem, Israel; ^29^ UO Gynecology Fondazione IRCCS San Gerardo dei Tintori, Monza, Italy; ^30^ Department of Medicine and Surgery, University of Milan-Bicocca, Milan, Italy; ^31^ Stroll Cancer Prevention Centre, Jewish General Hospital, and McGill University Medical School, Montreal, Canada; ^32^ Medical Oncology Department, Breast Unit, Fondazione IRCCS Istituto Nazionale dei Tumori, Milano, Italy; ^33^ Department of Oncology, Mayo Clinic College of Medicine, Rochester, MN; ^34^ Dipartimento di Scienze Chirurgiche, Oncologiche e Gastroenterologiche, Università di Padova, Padova, Italy; ^35^ Oncologia 2, Istituto Oncologico Veneto IOV-IRCCS, Padova, Italy; ^36^ Department of Oncology/Pathology, Karolinska Institute and Breast Center, Karolinska University Hospital, Stockholm, Sweden; ^37^ Smilow Cancer Hospital at Yale New Haven, New Haven, CT; ^38^ Breast Cancer Center, Hospital Zambrano Hellion—TecSalud, Tecnologico de Monterrey, Monterrey, Mexico; ^39^ Division of Medical Oncology, Department of Radiological, Oncological and Pathological Sciences, “La Sapienza” University of Rome, Rome, Italy; ^40^ Department of Hematology, Oncology and Dermatology, Umberto 1 University Hospital, Rome, Italy; ^41^ Department of Medical Oncology, Centro di Riferimento Oncologico di Aviano (CRO) IRCCS, Aviano, Italy; ^42^ Department of Medicine, University of Udine, Udine, Italy; ^43^ Karolinska Institutet, Department of Oncology-Pathology, Laboratory of Translational Fertility Preservation, New Karolinska Hospital, ME Gynecology and Reproduction, Stockholm, Sweden; ^44^ Department of Medical Oncology, King Albert II Cancer Institute, Cliniques Universitaires Saint-Luc and Institut de Recherche Expérimentale et Clinique (Pôle MIRO), UCLouvain, Brussels, Belgium; ^45^ Department of Medical Oncology, Hospital of Prato, Azienda USL Toscana Centro, Prato, Italy; ^46^ Hereditary Cancer Genetics Unit, Medical Oncology Department, Vall d'Hebron University Hospital, Vall d'Hebron Institute of Oncology (VHIO), Barcelona, Spain

## Abstract

**PURPOSE:**

To investigate the clinical behavior of breast cancer in young *BRCA* carriers according to the specific *BRCA* gene (*BRCA1 v BRCA2*) and the association of the timing of genetic testing (before *v* at diagnosis) with prognosis.

**METHODS:**

This was an international, multicenter, hospital-based, retrospective cohort study that included 4,752 patients harboring germline pathogenic/likely pathogenic variants (PVs) in *BRCA1* or *BRCA2*, who were diagnosed with stage I-III invasive breast cancer at 40 years or younger between January 2000 and December 2020 in 78 centers worldwide (ClinicalTrials.gov identifier: NCT03673306).

**RESULTS:**

Compared with *BRCA2* carriers (n = 1,683), *BRCA1* carriers (n = 3,069) had more frequently hormone receptor–negative (74.4% *v* 15.5%) and high-grade (77.5% *v* 49.1%) tumors. Similar outcomes were observed in *BRCA1* and *BRCA2* carriers but with a different pattern and risk of disease-free survival events over time. Compared with patients tested for *BRCA* at diagnosis (ie, between 2 months before and up to 6 months after diagnosis; n = 1,671), those tested before diagnosis (ie, any time up to 2 months before diagnosis; n = 411) had smaller tumors (T1: 61.3% *v* 32.4%), less nodal involvement (N0: 65.9% *v* 50.8%), less frequently received chemotherapy (84.4% *v* 92.9%), and axillary dissection (37.5% *v* 47.4%). Patients tested before diagnosis had better overall survival (OS; unadjusted hazard ratio [HR], 0.61 [95% CI, 0.40 to 0.92]); however, this result lost statistical significance after adjustment for potential confounders including tumor stage (adjusted HR, 0.74 [95% CI, 0.47 to 1.15]).

**CONCLUSION:**

This global study provides evidence on the different clinical behavior of breast cancer in young *BRCA1* and *BRCA2* carriers. Identifying a *BRCA* PV in healthy individuals was associated with earlier-stage breast cancer diagnosis and lower treatment burden, as well as better unadjusted OS.

## INTRODUCTION

Breast cancer diagnosed in women age 40 years or younger requires special multidisciplinary care given their age-related issues and needs.^1^ Among them, germline genetic testing plays a critical role considering that more than 10% of young women with breast cancer are expected to carry a germline pathogenic/likely pathogenic variant (PV) in the *BRCA* genes.^2,3^ In young women, germline genetic testing has clear clinical implications in terms of reproductive counseling,^4^ surveillance, and prevention strategies as well as therapeutic value once diagnosed with breast cancer.^5,6^

**CONTEXT**

**Key Objective**
To investigate the clinical behavior of breast cancer in young *BRCA1* versus *BRCA2* carriers and the association of prediagnostic awareness of germline *BRCA* status with prognosis.
**Knowledge Generated**
In this global study including 4,752 young *BRCA* carriers with breast cancer, distinct patient, tumor, and treatment characteristics and a different pattern and risk of disease-free survival events over time were observed between *BRCA1* and *BRCA2* carriers. The identification of carrying *BRCA* pathogenic/likely pathogenic variants in healthy individuals was associated with earlier-stage breast cancer diagnosis and lower treatment burden, as well as better unadjusted overall survival.
**Relevance *(K.D. Miller)***
Genetic testing, whether for women diagnosed with breast cancer or cascade testing (testing of potentially affected family members), remains underutilized. These results show that the power of genetic information to improve outcome should reinvigorate our efforts to offer testing broadly.*
*Relevance section written by *JCO* Senior Deputy Editor Kathy D. Miller, MD.

Breast cancers arising in *BRCA* carriers are characterized by unique biologic features and clinical behavior.^7,8^ Loss of function of BRCA1 and BRCA2 proteins leads to genomic instability that affects tumor biology and may also influence sensitivity to standard systemic therapies, subsequent prognosis,^9^ and reproductive outcomes.^10,11^ In young patients, while several studies have investigated potential differences in outcomes between *BRCA* carriers and those with sporadic disease,^3,12,13^ limited evidence exists on whether breast cancers in *BRCA1* or *BRCA2* carriers may differ in clinical behavior beyond differences in tumor biology.^14^ Dedicated efforts to dissect the potential different contribution of the specific altered *BRCA* gene in the clinical behavior of breast cancer are crucial to personalize patients' counseling on surveillance, prevention, treatment, and survivorship strategies.

Over the past decade, indications for and clinical implications of germline genetic testing have radically changed.^15^ Since the first International Consensus Conference for Breast Cancer in Young Women (BCY1) in November 2012, young age at breast cancer diagnosis is considered per se a criterion for referring patients to genetic counseling irrespective of family history or tumor biology.^16^ Awareness of a germline *BRCA* PV is critical, especially among young women. No breast cancer screening is recommended below age 40 years for women with average risk of breast cancer.^17^ Conversely, women at higher-than-average risk including *BRCA* carriers are candidates for an intensive surveillance starting at age 25-30 years.^5,18^ Nevertheless, despite the known beneficial effect of screening for early diagnosis in *BRCA* carriers,^5,18^ limited evidence exists on the association of prediagnostic awareness of germline *BRCA* status with prognosis,^19-22^ and there are no specific data in young women with breast cancer.

The BRCA BCY Collaboration (ClinicalTrials.gov identifier: NCT03673306) is the largest global cohort of *BRCA* carriers with diagnosis of breast cancer at young age.^11^ Hence, this study represents a unique real-world cohort to explore the clinical behavior of breast cancer in young *BRCA1* and *BRCA2* carriers separately and the association of the timing of genetic testing with prognosis.

## METHODS

### Study Design and Participants

This was an international, multicenter, hospital-based retrospective cohort study conducted at 78 institutions worldwide. As previously reported,^11^ the study included women diagnosed with invasive breast cancer at age 40 years or younger between January 2000 and December 2020 and known to carry a germline PV in the *BRCA1* and/or *BRCA2* genes. For the present analysis, patients carrying PVs in both *BRCA1* and *BRCA2* genes and those known to carry a *BRCA* PV but unknown information if in the *BRCA1* or *BRCA2* gene were excluded.

Each participating institution performed diagnostic, staging, treatment, and follow-up procedures according to local clinical practice.

Genetic testing and pathologic examination were performed locally. Hormone receptor positivity was defined by the presence of estrogen and/or progesterone receptors in at least 1% of invasive tumor cells (10% for nine centers), as determined by immunostaining. Human epidermal growth factor receptor 2 (HER2) positivity was defined as an immunohistochemical score of 3+ or 2+ with gene amplification detected by in situ hybridization techniques.

Institut Jules Bordet in Brussels (Belgium) served as the central ethics committee. In compliance with the regulatory requirements of participating centers, the study received ethical approval from the local, regional, or national institutional review boards whenever required.

The reporting of the study followed the Strengthening the Reporting of Observational Studies in Epidemiology statement.^23^

### Outcomes

The objectives of this analysis were to explore the clinical behavior and outcomes of breast cancer in young *BRCA* carriers according to the specific *BRCA* gene (*BRCA1 v BRCA2*) and to assess the association of the timing of genetic testing (before *v* diagnosis) with prognosis.

For the first objective, all patients eligible for the present analysis were included and two groups were identified: women with *BRCA1* PVs (*BRCA1* carriers) and those with *BRCA2* PVs (*BRCA2* carriers). Clinicopathologic and treatment characteristics as well as survival outcomes were compared between *BRCA1* and *BRCA2* carriers. Subgroup analyses according to hormone receptor status were performed. To account for the potential lead time bias, sensitivity analyses were conducted by including only patients with *BRCA* testing performed any time up to 2 months before diagnosis of breast cancer (*BRCA* test-before-diagnosis group) and women tested from up to 2 months before and within 6 months after diagnosis of breast cancer (*BRCA* test-at-diagnosis group).

For the second objective, the comparison was made according to the timing of the test by including only patients in the *BRCA* test-before-diagnosis group and women in the *BRCA* test-at-diagnosis group. Patients with unknown date of *BRCA* testing and those tested during follow-up were excluded from this analysis. Subgroup analyses according to the specific *BRCA* gene were performed.

### Statistical Analysis

Descriptive analyses were used to compare clinicopathologic and treatment characteristics. The Chi-Square test and Wilcoxon test were used to compare categorical and continuous variables as appropriate. For survival analyses, the following end points were considered and defined as previously reported^11^: disease-free survival (DFS), breast cancer–specific survival (BCSS), and overall survival (OS). For patients who did not encounter an event, observation times were censored at the date of their last contact. For the first objective, all eligible patients were included and sensitivity analyses including only those who tested before and at diagnosis were performed. For the second objective, only patients tested before and at diagnosis were included.

Rates for DFS events were computed as the ratio between the total number of events and the total of the observation times. To assess the pattern of DFS events over time, the Epanechnikov Kernel-Smoothed annual hazard of DFS events was computed. The optimal width of the density window in the Kernel-smoothed estimates was selected to minimize the mean-integrated squared error. The number of points for density estimation was set to 50. Kaplan-Meier plots were used to illustrate results with a follow-up period up to 15 years. The Cox proportional hazard model was applied to estimate hazard ratios (HRs), while adjusting for the concurrent effect of selected confounders. Before applying Cox proportional hazard models, visual inspection of the plots of Schoenfeld residuals and Grambsch-Therneau test was performed. In case of violation of the proportional hazard assumption, Cox models were not performed. When the proportional hazard assumption was fulfilled, multivariate models for survival analyses incorporated factors that were known to be prognostic or were differently distributed between the two groups (ie, country, year at diagnosis, specific *BRCA* gene, grade, tumor size, nodal status, axillary surgery, and chemotherapy use). Country and year at diagnosis were included in the models as stratification factors, whereas specific *BRCA* gene, grade, tumor size, nodal status, axillary surgery, and chemotherapy use were included as covariates. No imputation methods were used to handle missing values that were included in all models as a separate category.

All statistical analyses were two-sided, with *P* < .05 considered statistically significant. No adjustment for multiplicity was performed. The analyses were performed using Stata, software version 16.1 (StataCorp LLC, College Station, TX).

## RESULTS

### *BRCA1* Versus *BRCA2*

**FIG 1.**
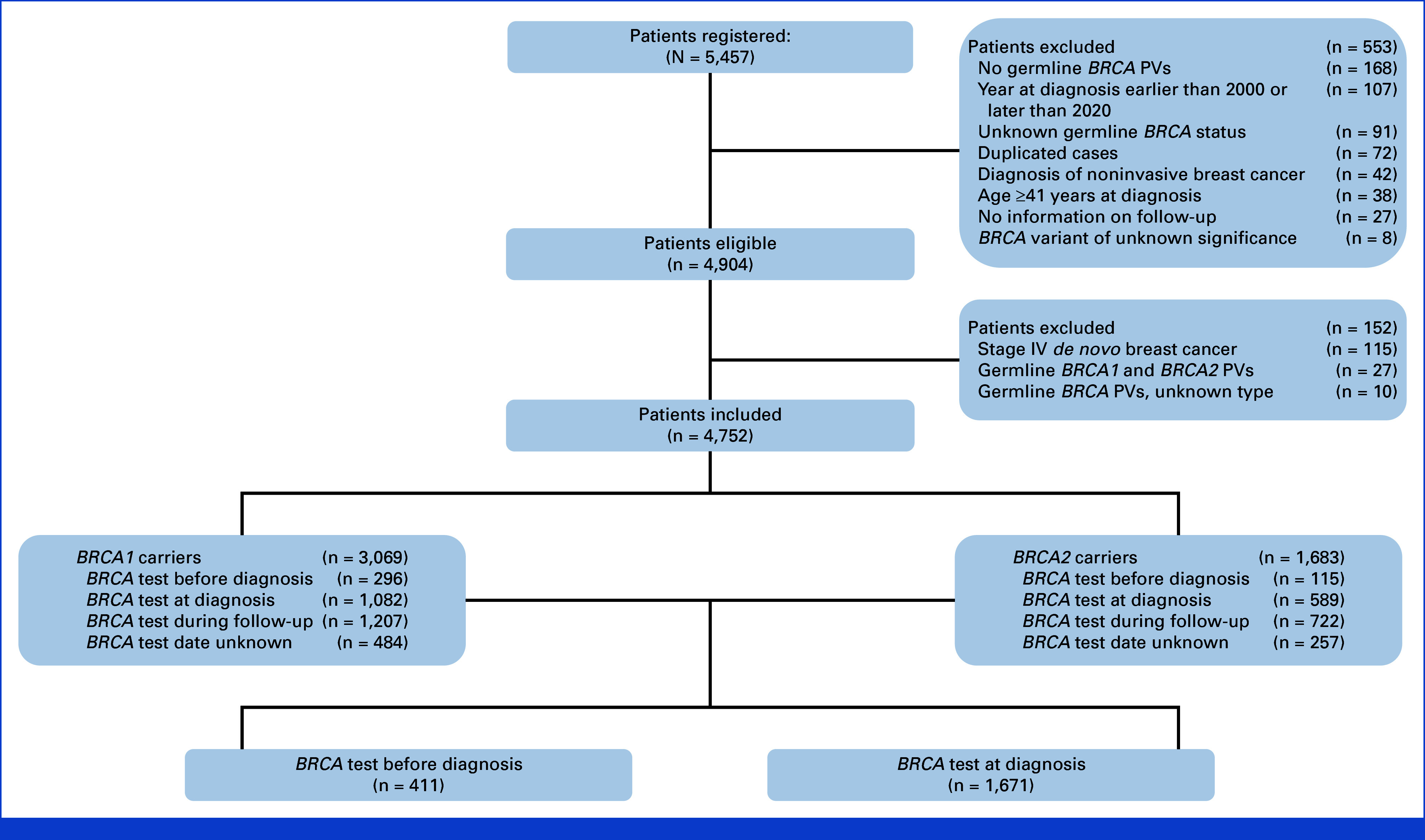
Study flowchart. PV, pathogenic/likely pathogenic variant.

A total of 4,752 young women with breast cancer were included in the present analysis, of whom 3,069 were *BRCA1* carriers and 1,683 were *BRCA2* carriers (Fig 1).

Compared with patients in the *BRCA2* group, *BRCA1* carriers were younger at diagnosis (median age, 34 [IQR, 31-37] *v* 35 [IQR, 32-38] years) and had more frequently hormone receptor–negative (74.4% *v* 15.5%) and high-grade (77.5% *v* 49.1%) tumors, fewer small tumors (T1: 37.1% *v* 40.5%), less nodal involvement (N0: 56.7% *v* 41.6%), lobular histology (1.2% *v* 5.7%), and HER2 positivity (4.8% *v* 11.2%; Table 1). In *BRCA1* carriers, chemotherapy was administered more frequently (94.3% *v* 85.4%) than in *BRCA2* carriers; in the case of hormone receptor–positive disease, endocrine therapy was received less often (89.3% *v* 95.5%). Radical mastectomy was the most common surgical treatment in both patient groups; however, breast-conserving surgery was more frequently performed in *BRCA1* than in *BRCA2* carriers (42.9% *v* 30.6%). Axillary dissection was less commonly performed in *BRCA1* than in *BRCA2* carriers (46.9% *v* 58.6%). A total of 1,753 (57.1%) *BRCA1* carriers and 949 (56.4%) *BRCA2* carriers underwent risk-reducing mastectomy, whereas 1,591 (51.8%) *BRCA1* carriers and 851 (50.6%) *BRCA2* carriers underwent risk-reducing salpingo-oophorectomy during follow-up. Patient, tumor, and treatment characteristics at the time of breast cancer diagnosis in *BRCA1* versus *BRCA2* carriers according to the timing of germline *BRCA* testing are reported in the Data Supplement (Table S1, online only).

**TABLE 1.** Patient, Tumor, and Treatment Characteristics According to the Specific *BRCA* Gene

Variable	*BRCA1* Carriers (n = 3,069)	*BRCA2* Carriers (n = 1,683)	*P* ^a^
Country, No. (%)			<.001
North America	324 (10.6)	193 (11.5)	
South-Center America	105 (3.4)	41 (2.4)	
Asia + Israel	539 (17.6)	235 (14.0)	
Oceania	114 (3.7)	84 (5.0)	
North Europe	470 (15.3)	250 (14.8)	
South Europe	1,278 (41.6)	810 (48.1)	
East Europe	239 (7.8)	70 (4.2)	
Year at diagnosis, No. (%)			.370
2000-2005	485 (15.8)	275 (16.3)	
2006-2010	745 (24.3)	391 (23.2)	
2011-2015	891 (29.0)	462 (27.5)	
2016-2020	948 (30.9)	555 (33.0)	
Age at diagnosis, years, median (IQR)	34 (31-37)	35 (32-38)	.003
Age at diagnosis, years, No. (%)			<.001
≤30	705 (23.0)	272 (16.2)	
31-35	1,088 (35.4)	636 (37.8)	
36-40	1,276 (41.6)	775 (46.0)	
Time from diagnosis to *BRCA* testing, months, median (IQR)	5.3 (0.8-24.3)	5.9 (1.0-28.1)	.062
Missing, No.	484	257	
Histology, No. (%)			<.001
Ductal carcinoma	2,606 (84.9)	1,335 (79.3)	
Lobular carcinoma	38 (1.2)	96 (5.7)	
Invasive, not specified	130 (4.2)	69 (4.1)	
Others	195 (6.3)	124 (7.4)	
Missing	100 (3.3)	59 (3.5)	
Tumor grade, No. (%)			<.001
G1	23 (0.7)	56 (3.3)	
G2	395 (12.9)	602 (35.8)	
G3	2,378 (77.5)	827 (49.1)	
Missing	273 (8.9)	198 (11.8)	
Tumor size, No. (%)			.001
T1	1,138 (37.1)	681 (40.5)	
T2	1,385 (45.1)	662 (39.3)	
T3-T4	396 (12.9)	244 (14.5)	
Missing	150 (4.9)	96 (5.7)	
Nodal status, No. (%)			<.001
N0	1,741 (56.7)	701 (41.6)	
N1	919 (29.9)	640 (38.0)	
N2-N3	296 (9.6)	258 (15.3)	
Missing	113 (3.7)	84 (5.0)	
Hormone receptor status, No. (%)			<.001
ER- and/or PR-positive	736 (24.0)	1,394 (82.8)	
ER- and PR-negative	2,282 (74.4)	261 (15.5)	
Missing	51 (1.7)	28 (1.7)	
HER2 status, No. (%)			<.001
HER2-negative	2,776 (90.4)	1,398 (83.1)	
HER2-positive	147 (4.8)	188 (11.2)	
Missing	146 (4.8)	97 (5.8)	
Breast surgery, No. (%)			<.001
Not performed	9 (0.3)	6 (0.4)	
Breast-conserving surgery	1,317 (42.9)	515 (30.6)	
Mastectomy	1,680 (54.7)	1,124 (66.8)	
Missing	63 (2.0)	38 (2.3)	
Axillary surgery, No. (%)			<.001
Not performed	53 (1.7)	40 (2.4)	
Sentinel node biopsy only	1,346 (43.9)	585 (34.8)	
Axillary dissection	1,440 (46.9)	987 (58.6)	
Missing	230 (7.5)	71 (4.2)	
Use of chemotherapy, No. (%)			<.001
No	153 (5.0)	231 (13.7)	
Yes	2,895 (94.3)	1,437 (85.4)	
Missing	21 (0.7)	15 (0.9)	
Type of chemotherapy,^b^ No. (%)			.003
Anthracycline- and taxane-based	2,047 (70.7)	1,010 (70.3)	
Anthracycline-based	540 (18.6)	258 (17.9)	
Taxane-based	112 (3.9)	75 (5.2)	
Others	103 (3.6)	27 (1.9)	
Missing	93 (3.2)	67 (4.7)	
Timing of chemotherapy administration,^b^ No. (%)			.003
Neoadjuvant	1,370 (47.3)	613 (42.7)	
Adjuvant	1,509 (52.1)	820 (57.1)	
Missing	16 (0.6)	4 (0.3)	
Use of endocrine therapy,^c^ No. (%)			<.001
No	71 (9.6)	41 (2.9)	
Yes	657 (89.3)	1,332 (95.5)	
Missing	8 (1.1)	21 (1.5)	
Type of endocrine therapy,^d^ No. (%)			.026
Tamoxifen alone	250 (38.0)	457 (34.3)	
Tamoxifen + LHRHa	167 (25.4)	384 (28.8)	
LHRHa alone	21 (3.2)	20 (1.5)	
AI with or without LHRHa	111 (16.9)	242 (18.2)	
Tamoxifen and AI (with or without LHRHa)	88 (13.4)	203 (15.2)	
Others	12 (1.8)	14 (1.0)	
Missing	8 (1.2)	12 (0.9)	
Duration of endocrine therapy, months, median (IQR)	58 (24-60)	60 (28.5-60)	.470
Missing, No.	186	320	

Abbreviations: AI, aromatase inhibitors; ER, estrogen receptor; G, tumor grade; HER2, human epidermal growth factor receptor 2; LHRHa, luteinizing hormone-releasing hormone agonists; N, nodal status; PR, progesterone receptor; T, tumor size.

^a^ Calculated after exclusion of missing values.

^b^ Calculated among patients who received chemotherapy.

^c^ Calculated among patients with hormone receptor–positive breast cancer.

^d^ Calculated among patients with hormone receptor–positive breast cancer who received endocrine therapy.

At a median follow-up of 7.8 years (IQR, 4.4-12.6 years), 1,691 DFS events were observed (Data Supplement, Table S2). Second primary breast cancers (2.12 *v* 1.42 events per 100 person-year) and nonbreast primary malignancies (0.70 *v* 0.45 events per 100 person-year) were more frequent among *BRCA1* than *BRCA2* carriers, whereas distant recurrences were less frequent (1.51 *v* 2.06 events per 100 person-year).

**FIG 2.**
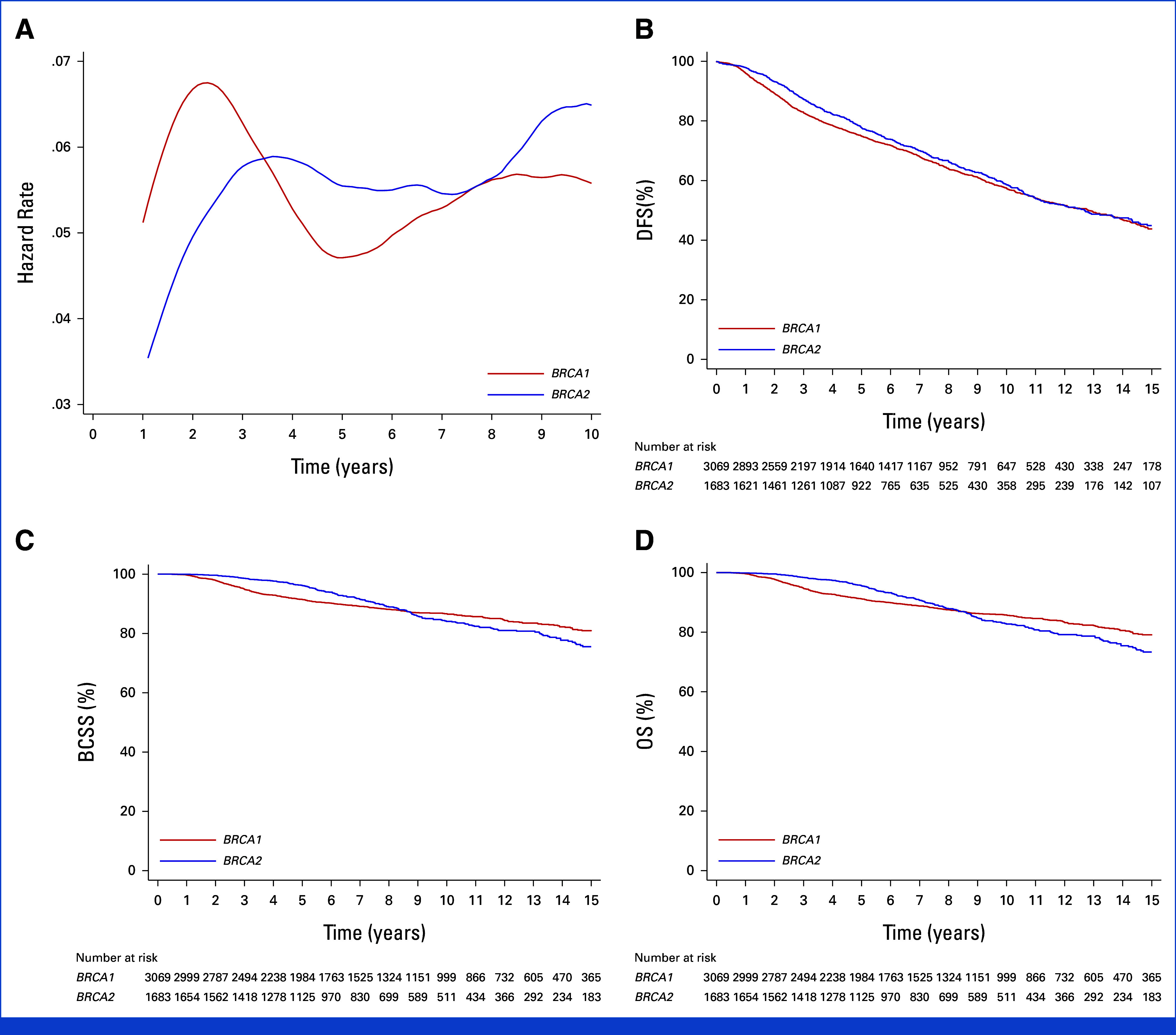
Survival outcomes in *BRCA1* and *BRCA2* carriers: (A) Epanechnikov Kernel-Smoothed annual hazard of DFS events overall, (B) DFS, (C) BCSS, and (D) OS. BCSS, breast cancer–specific survival; DFS, disease-free survival; OS, overall survival.

When considering timing of DFS events, the hazard rate over time in *BRCA1* carriers was higher during the first 2 years and then declined until year 6, at which point there was a new increase in risk. In *BRCA2* carriers, the hazard rate progressively increased during the first 3 years before stabilizing and remaining constant in the following years (Fig 2A).

The 8-year DFS was 63.8% (95% CI, 61.8 to 65.8) for *BRCA1* and 66.2% (95% CI, 63.5 to 68.9) for *BRCA2* carriers (Fig 2B). *BRCA1* carriers had a higher risk of BCSS and OS events during the first 8 years after diagnosis, whereas the risk was greater for *BRCA2* carriers afterward (the 8-year BCSS was 88.1%; 95% CI, 86.7 to 89.4 for *BRCA1* and 88.9%; 95% CI, 86.9 to 90.7 for *BRCA2* carriers; the 8-year OS was 87.5%; 95% CI, 86.1 to 88.8 for *BRCA1* and 87.9%; 95% CI, 85.8 to 89.7 for *BRCA2* carriers; Figs 2C and 2D). For all survival end points, violation of the proportional hazard assumption occurred.

To account for the potential lead time bias, sensitivity analyses comparing *BRCA1* versus *BRCA2* carriers were repeated by including only patients tested before or at diagnosis. Results were superimposable with those observed in the entire cohort (Data Supplement, Tables S3 and S4 and Fig S1).

Tumor and treatment characteristics in *BRCA1* and *BRCA2* carriers according to hormone receptor status are reported in the Data Supplement (Table S5), and those according to the type of first DFS events are reported in the Data Supplement (Table S6). Consistent DFS, BCSS, and OS results as in the entire cohort were observed between *BRCA1* and *BRCA2* carriers with hormone receptor–positive (Data Supplement, Fig S2) and hormone receptor–negative breast cancers (Data Supplement, Fig S3).

### *BRCA* Test Before Diagnosis Versus *BRCA* Test at Diagnosis

Among 4,011 patients with the known date of germline *BRCA* testing, 411 were tested before diagnosis and 1,671 were tested at diagnosis (Fig 1).

Compared with the *BRCA* test-at-diagnosis group, those who underwent genetic testing before diagnosis had smaller tumors (T1: 61.3% *v* 32.4%) and less nodal involvement (N0: 65.9% *v* 50.8%; Table 2). Chemotherapy was administered less frequently in patients tested before diagnosis (84.4% *v* 92.9%); among women receiving chemotherapy, fewer patients in the *BRCA* test-before-diagnosis group were treated in the neoadjuvant setting (38.0% *v* 57.7%), whereas a higher number of them were exposed to an anthracycline-free taxane-based regimen (8.4% *v* 4.4%). Axillary dissection was less frequently performed in patients in the *BRCA* test-before-diagnosis group (37.5% *v* 47.4%). A total of 323 (78.6%) and 1,059 (63.4%) patients in the *BRCA* test-before- and *BRCA* test-at-diagnosis groups underwent risk-reducing mastectomy, whereas 229 (55.7%) and 831 (49.7%) underwent risk-reducing salpingo-oophorectomy during study follow-up.

**TABLE 2.** Patient, Tumor, and Treatment Characteristics in Patients Who Underwent Germline *BRCA* Testing Before and at Breast Cancer Diagnosis

Variable	*BRCA* Test Before Diagnosis (n = 411)	*BRCA* Test at Diagnosis (n = 1,671)	*P* ^a^
Country, No. (%)			<.001
North America	50 (12.2)	191 (11.4)	
South-Center America	1 (0.2)	20 (1.2)	
Asia + Israel	85 (20.7)	362 (21.7)	
Oceania	34 (8.3)	57 (3.4)	
North Europe	62 (15.1)	240 (14.4)	
South Europe	155 (37.7)	660 (39.5)	
East Europe	24 (5.8)	141 (8.4)	
Year at diagnosis, No. (%)			.784
2000-2005	20 (4.9)	87 (5.2)	
2006-2010	69 (16.8)	268 (16.0)	
2011-2015	129 (31.4)	490 (29.3)	
2016-2020	193 (47.0)	826 (49.4)	
Age at diagnosis, years, median (IQR)	35 (31-38)	35 (31-38)	.469
Age at diagnosis, years, No. (%)			.375
≤30	93 (22.6)	350 (20.9)	
31-35	136 (33.1)	614 (36.7)	
36-40	182 (44.3)	707 (42.3)	
Specific *BRCA* gene, No. (%)			.005
*BRCA1* carriers	296 (72.0)	1,082 (64.8)	
*BRCA2* carriers	115 (28.0)	589 (35.2)	
Histology, No. (%)			.180
Ductal carcinoma	354 (86.1)	1,444 (86.4)	
Lobular carcinoma	15 (3.6)	37 (2.2)	
Invasive, not specified	25 (6.1)	90 (5.4)	
Others	16 (3.9)	94 (5.6)	
Missing	1 (0.2)	6 (0.4)	
Tumor grade, No. (%)			<.001
G1	16 (3.9)	18 (1.1)	
G2	76 (18.5)	361 (21.6)	
G3	291 (70.8)	1,103 (66.0)	
Missing	28 (6.8)	189 (11.3)	
Tumor size, No. (%)			<.001
T1	252 (61.3)	541 (32.4)	
T2	118 (28.7)	803 (48.1)	
T3-T4	28 (6.8)	271 (16.2)	
Missing	13 (3.2)	56 (3.3)	
Nodal status, No. (%)			<.001
N0	271 (65.9)	849 (50.8)	
N1	95 (23.1)	574 (34.3)	
N2-N3	35 (8.5)	216 (12.9)	
Missing	10 (2.4)	32 (1.9)	
Hormone receptor status, No. (%)			.231
ER- and/or PR-positive	170 (41.4)	752 (45.0)	
ER- and PR-negative	237 (57.7)	917 (54.9)	
Missing	4 (1.0)	2 (0.1)	
HER2 status, No. (%)			.226
HER2-negative	382 (92.9)	1,540 (92.2)	
HER2-positive	20 (4.9)	109 (6.5)	
Missing	9 (2.2)	22 (1.3)	
Breast surgery, No. (%)			.566
Not performed	1 (0.2)	7 (0.4)	
Breast-conserving surgery	113 (27.5)	498 (29.8)	
Mastectomy	294 (71.5)	1,155 (69.1)	
Missing	3 (0.7)	11 (0.7)	
Axillary surgery, No. (%)			<.001
Not performed	7 (1.7)	26 (1.6)	
Sentinel node biopsy only	238 (57.9)	785 (47.0)	
Axillary dissection	154 (37.5)	792 (47.4)	
Missing	12 (2.9)	68 (4.1)	
Use of chemotherapy, No. (%)			<.001
No	61 (14.8)	111 (6.6)	
Yes	347 (84.4)	1,552 (92.9)	
Missing	3 (0.7)	8 (0.5)	
Type of chemotherapy,^b^ No. (%)			.023
Anthracycline- and taxane-based	267 (76.9)	1,222 (78.7)	
Anthracycline-based	37 (10.7)	197 (12.7)	
Taxane-based	29 (8.4)	69 (4.4)	
Others	7 (2.0)	27 (1.7)	
Missing	7 (2.0)	37 (2.4)	
Timing of chemotherapy administration,^b^ No. (%)			<.001
Neoadjuvant	132 (38.0)	896 (57.7)	
Adjuvant	214 (61.7)	651 (42.0)	
Missing	1 (0.3)	5 (0.3)	
Use of endocrine therapy,^c^ No. (%)			.219
No	13 (7.6)	39 (5.2)	
Yes	155 (91.2)	698 (92.8)	
Missing	2 (1.2)	15 (2.0)	
Type of endocrine therapy,^d^ No. (%)			.069
Tamoxifen alone	56 (36.1)	201 (28.8)	
Tamoxifen + LHRHa	29 (18.7)	191 (27.4)	
LHRHa alone	5 (3.2)	13 (1.9)	
AI with or without LHRHa	43 (27.7)	160 (22.9)	
Tamoxifen and AI (with or without LHRHa)	18 (11.6)	115 (16.5)	
Others	3 (1.9)	13 (1.9)	
Missing	1 (0.6)	5 (0.7)	
Duration of endocrine therapy, months, median (IQR)	38.5 (24-60)	49.5 (24-60)	.299
Missing, No.	43	164	

Abbreviations: AI, aromatase inhibitors; ER, estrogen receptor; G, tumor grade; HER2, human epidermal growth factor receptor 2; LHRHa, luteinizing hormone-releasing hormone agonists; N, nodal status; PR, progesterone receptor; T, tumor size.

^a^Calculated after exclusion of missing values.

^b^ Calculated among patients who received chemotherapy.

^c^ Calculated among patients with hormone receptor–positive breast cancer.

^d^ Calculated among patients with hormone receptor–positive breast cancer who received endocrine therapy.

**FIG 3.**
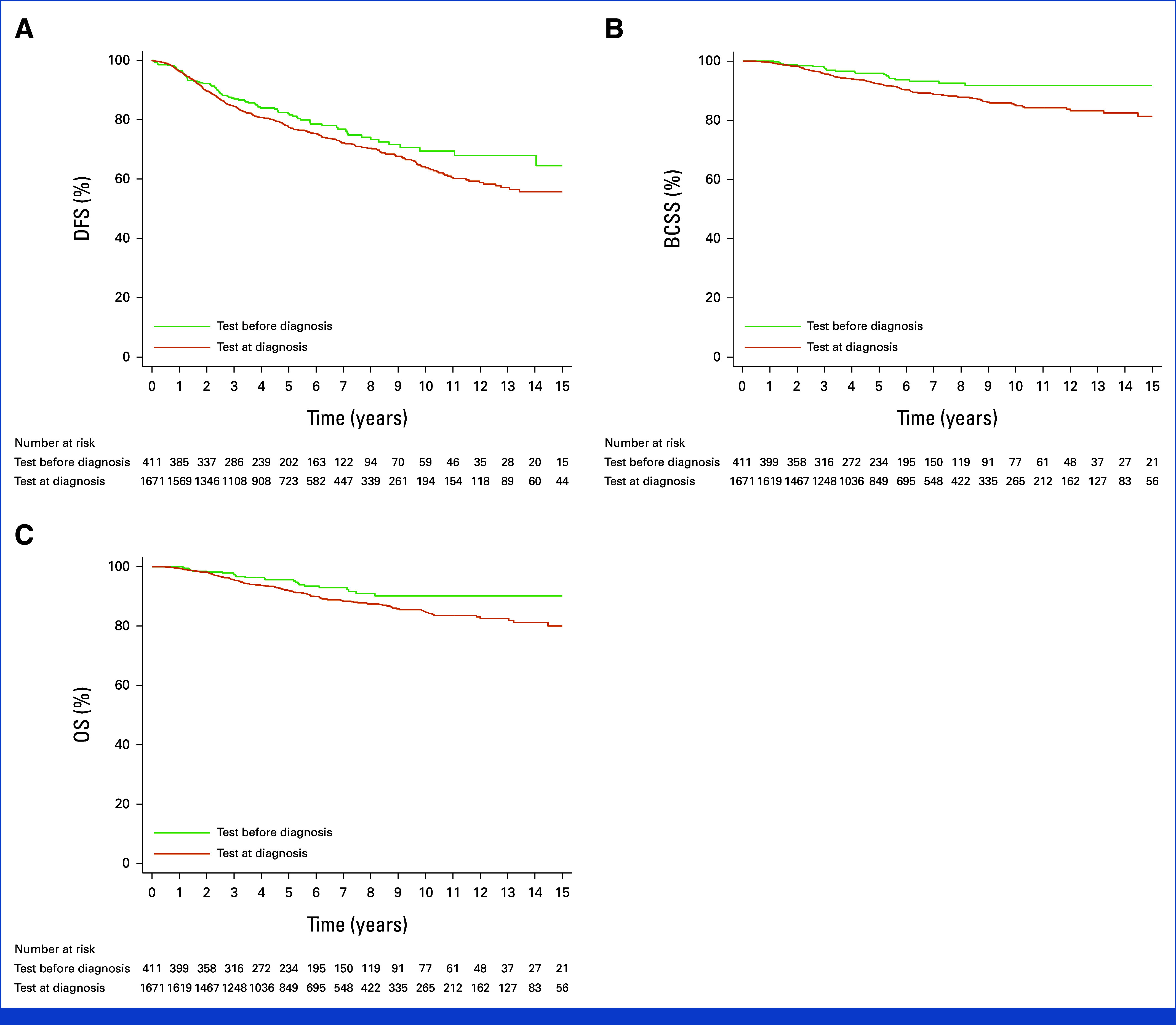
Survival outcomes in patients tested for germline *BRCA* status before or at diagnosis of breast cancer: (A) DFS, (B) BCSS, and (C) OS. BCSS, breast cancer–specific survival; DFS, disease-free survival; OS, overall survival.

The type of DFS events according to the timing of *BRCA* testing is reported in the Data Supplement (Table S7). The 8-year DFS was 73.3% (95% CI, 67.3 to 78.4) in the *BRCA* test-before-diagnosis group and 70.4% (95% CI, 67.5 to 73.1) in the *BRCA* test-at-diagnosis group (unadjusted HR, 0.80 [95% CI, 0.63 to 1.01]; adjusted HR, 0.91 [95% CI, 0.71 to 1.16]; Fig 3A; Data Supplement, Table S8). The 8-year BCSS was 92.5% (95% CI, 88.6 to 95.2) and 87.8% (95% CI, 85.6 to 89.7) in the *BRCA* test-before- and *BRCA* test-at-diagnosis groups, respectively (unadjusted HR, 0.56 [95% CI, 0.36 to 0.87]; adjusted HR, 0.68 [95% CI, 0.43 to 1.09]; Fig 3B; Data Supplement, Table S8). The 8-year OS was 90.7% (95% CI, 86.5 to 94.0) in the *BRCA* test-before-diagnosis group and 87.4% (95% CI, 85.2 to 89.4) in the *BRCA* test-at-diagnosis group (unadjusted HR, 0.61 [95% CI, 0.40 to 0.92]; adjusted HR, 0.74 [95% CI, 0.47 to 1.15]; Fig 3C; Data Supplement, Table S8).

Patient, tumor, and treatment characteristics of patients who underwent germline *BRCA* testing before and at breast cancer diagnosis according to the specific *BRCA* gene are reported in the Data Supplement (Table S9), and the type of DFS event are reported in the Data Supplement (Table S10). A significant interaction between specific *BRCA* gene and timing of *BRCA* testing was observed in DFS (*P* for interaction = .010), whereas similar results as in the entire cohort were observed in BCSS and OS (Data Supplement, Table S11 and Fig S4).

## DISCUSSION

In this global study of young *BRCA* carriers with breast cancer, distinct patient, tumor, and treatment characteristics and a different pattern and risk of survival events over time were observed between patients carrying germline *BRCA1* and *BRCA2* PVs. Identification of carrying a *BRCA* PV in healthy individuals was associated with earlier-stage breast cancer diagnosis and lower treatment burden, as well as better unadjusted OS.

In patients with breast cancer, the specific altered *BRCA* gene is known to be associated with different clinicopathologic features, with the majority of tumors being triple-negative in *BRCA1* carriers and hormone receptor–positive/HER2-negative in *BRCA2* carriers.^7,8,24,25^ These peculiar biologic features were also observed in our study. Notably, different from the frequency of germline *BRCA1* and *BRCA2* PVs observed in population-based studies,^26^ the majority of patients in our study were *BRCA1* carriers. This result may be explained by the specific patient population that we included considering the higher risk of developing breast cancer at a young age in *BRCA1* carriers^27^ and the increased likelihood of developing triple-negative disease in young women.^28^

In terms of prognosis, current evidence does not support different survival outcomes between patients with sporadic disease and *BRCA* carriers.^29^ Notably, most studies investigating this issue considered all *BRCA* carriers without differentiating according to the specific altered *BRCA* gene, or when considering *BRCA1* and *BRCA2* carriers separately, a comparison between them was often not performed or analyses were underpowered. In our study of young women with breast cancer, although there were no apparent differences in survival outcomes between *BRCA1* and *BRCA2* carriers, a distinct pattern of DFS events over time was observed with a peak among *BRCA1* carriers in the first 2 years and a constant risk over time in *BRCA2* carriers that led to worse long-term OS. This pattern may be explained by the different distribution of breast cancer subtypes in *BRCA1* and *BRCA2* carriers.^30^ Notably, these outcomes should be interpreted in the context of the systemic therapy received by the patients (of whom 91.2% received chemotherapy, with modern anthracycline- and taxane-based regimens in 70% of the cases and use of ovarian function suppression in 62.1% of carriers with hormone receptor–positive disease). During the period of eligibility to the study, immunotherapy, adjuvant olaparib, or CDK4/6 inhibitors were not yet standard of care.

The differential role of *BRCA1* and *BRCA2* PVs in the age-related risk of developing breast cancer and other malignancies is well established,^27^ with subsequent distinct recommendations for surveillance and prevention strategies.^5^ Moreover, age <40 years at primary diagnosis is a known risk factor for cumulative risk of contralateral breast cancer, particularly among *BRCA1* carriers.^31^ Our findings showed that the specific altered *BRCA* gene may be associated with different age at breast cancer onset and type of first DFS event. As compared with *BRCA2* carriers, patients with *BRCA1* PVs were younger at diagnosis and more often developed second primary breast and nonbreast malignancies. These data raise awareness on the importance of developing tailored surveillance, prevention, and follow-up strategies for patients with hereditary breast cancer that should consider both age at first diagnosis and the specific altered *BRCA* gene. Future efforts in clinical trials including *BRCA* carriers should be made to report outcomes and treatment effects separately in patients with *BRCA1* and *BRCA2* PVs and to record surveillance and prevention strategies of those who are further under study.

In the past few years, the indications for germline genetic testing in patients with breast cancer have remarkably expanded.^6^ The recommended intensive surveillance in healthy *BRCA* carriers leads to earlier breast cancer diagnosis^19-21,32,33^ and is cost-effective.^34^ However, very limited information exists on the impact of germline testing on oncologic outcomes,^19-22^ with no evidence in the specific cohort of young women. In our study, patients known to carry a *BRCA1* or *BRCA2* PV before diagnosis were diagnosed more often with T1 tumors and node-negative disease as compared with those who were tested after diagnosis and underwent less frequently axillary dissection and chemotherapy. Importantly, knowledge of *BRCA* status before breast cancer diagnosis was associated with a trend toward improved DFS (in *BRCA1* carriers only) and significantly better unadjusted BCSS and OS (in both *BRCA1* and *BRCA2* carriers). Although information on prevention strategies was not collected in our study, these data may suggest that awareness of carrying a *BRCA* PV before diagnosis was likely associated with enhanced surveillance and increased health care–seeking behaviors among *BRCA* carriers. As a consequence, this attitude could explain the observed breast cancer downstaging and its subsequent downstream benefits including less aggressive surgical and systemic treatments. The lack of statistical significance observed in the multivariate models may indicate that timing of *BRCA* test itself did not influence prognosis but that the observed survival differences were likely explained by different tumor features including more advanced stage in patients tested at diagnosis. With improved knowledge of breast cancer biology and the availability of biomarkers for refining chemotherapy indications,^35,36^ future research efforts are needed to optimize the systemic treatment particularly among patients with stage I disease (of whom 79.7% received chemotherapy in our study).

In addition to its retrospective nature, other limitations of the study include that *BRCA* genetic testing, determination of tumor characteristics, anticancer treatments, and follow-up were performed locally according to standard practice. The study was conducted in 78 different centers from 26 countries in four continents over a 20-year time frame. Hence, different health care systems and changes in practice during the study period might have influenced the results. Information on prevention strategies before breast cancer diagnosis was not collected. Moreover, it is not possible to exclude the fact that other unmeasured differences might have contributed to the survival results according to the timing of genetic testing, including greater health care–seeking behaviors in patients tested before diagnosis, which might have also led to better OS once diagnosed (ie, the healthy user effect). Finally, considering the nature of the study design and the absence of multiple testing adjustment, all analyses should be considered exploratory. However, the uniqueness of this cohort (including only young *BRCA* carriers with breast cancer), the global representation, and the relatively long follow-up are important strengths.

In conclusion, our global study including young *BRCA* carriers provides evidence on the different clinical behavior of breast cancer according to the specific *BRCA* gene and the association of the timing of genetic testing with prognosis. *BRCA1* and *BRCA2* carriers were characterized by distinct patient, tumor, and treatment characteristics and a different pattern and risk of DFS events over time. Identification of carrying a *BRCA* PV in healthy individuals was associated with earlier-stage breast cancer diagnosis and lower treatment burden, as well as better unadjusted OS. Increased awareness on the importance of identifying healthy women at risk of carrying a *BRCA1* or *BRCA2* PV is needed to offer genetic counseling and testing to inform them about early detection options that may lead to better prognosis.

## Data Availability

Data will be available for sharing with researchers who provide a methodologically sound proposal after proper revision of the data transfer agreement of each participating center and if ultimately allowed by the local ethics committee. The types of analyses allowed will be those able to achieve the aims of the approved proposal. Proposals should be directed to matteo.lambertini@unige.it.
